# MicroRNA-146a and Ets-1 Gene Polymorphisms Are Associated with Pediatric Uveitis

**DOI:** 10.1371/journal.pone.0091199

**Published:** 2014-03-21

**Authors:** Lin Wei, Qingyun Zhou, Shengping Hou, Lin Bai, Yunjia Liu, Jian Qi, Qin Xiang, Yan Zhou, Aize Kijlstra, Peizeng Yang

**Affiliations:** 1 The First Affiliated Hospital of Chongqing Medical University, Chongqing Key Laboratory of Ophthalmology and Chongqing Eye Institute, Chongqing, P. R. China; 2 University Eye Clinic Maastricht, Maastricht, The Netherlands; University of Miami School of Medicine, United States of America

## Abstract

**Background:**

MicroRNA-146a (miR-146a) was a key negative regulator of autoimmunity. V-Ets oncogene homolog 1 (Ets-1) was demonstrated to bind to the miR-146a promoter region and markedly affects miR-146a promoter activity. This study aimed to investigate the association of miR-146a and Ets-1 gene polymorphisms with pediatric uveitis in a Han Chinese population.

**Methodology/Principal Findings:**

A total of 520 patients and 1204 healthy controls were included in the present study. Five single-nucleotide polymorphisms (SNPs), miR-146a/rs2910164, miR-146a/rs57095329, miR-146a/rs6864584, ets-1/rs1128334 and ets-1/rs10893872 were genotyped using a polymerase chain reaction-restriction fragment length polymorphism assay. The expression of Ets-1 in peripheral blood mononuclear cells from genotyped healthy controls was tested by real-time PCR. Two SNPs (rs2910164 and rs10893872) were associated with pediatric uveitis in this study. The frequencies of the rs2910164 GG genotype and G allele were significantly increased (P_c_ = 3.11×10^−4^; P_c_ = 2.75×10^−6^) while the CC genotype and C allele were significantly decreased (P_c_ = 0.001; P_c_ = 2.75×10^−6^) in patients compared with normal controls. The frequencies of the rs10893872 CC genotype and C allele were significantly increased (P_c_ = 3.89×10^−4^; P_c_ = 0.01) while the CT genotype and T allele were significantly decreased (P_c_ = 0.004; P_c_ = 0.01) in patients compared with normal controls. The SNP rs2910164 GG genotype and G/C allele were also associated with the presence of microvascular leakage as detected by fundus fluorescein angiography in pediatric uveitis (P_c_ = 0.01; P_c_ = 0.005, respectively). Ets-1 expression in rs10893872 CC carriers was significantly higher than in CT and TT individuals (P_c_ = 0.013). There was no association of the other three SNPs with pediatric uveitis.

**Conclusions:**

This study shows that miR-146a and Ets-1 are both associated with pediatric uveitis in Han Chinese. SNP rs10893872 may affect the genetic predisposition to pediatric uveitis by modulating expression of Ets-1.

## Introduction

Uveitis is a potentially sight threatening disease and one of the important causes of blindness in the world. It may occur due to an infection or may be due to an autoimmune etiology [1]–[3]. Children account for 5%∼10% of all uveitis patients [4], [5]. Although uveitis is less common in children than in adults, patients with pediatric uveitis more often develop serious complications such as cataract and glaucoma that result in visual loss [4]. Epidemiologic studies indicate that idiopathic uveitis (28.8%) is the most common subtype of pediatric uveitis, followed by juvenile idiopathic arthritis (JIA)-associated uveitis (20.9%) and pars planitis (17.1%) [6]. Fundus fluorescein angiography (FFA), rheumatoid factor (RF) and anti-nuclear antibody (ANA) are considered as important markers for pediatric uveitis [7]. Genetic factors have been reported to be involved in the pathogenesis of pediatric uveitis and a study of 316 Caucasian children demonstrated that human leukocyte antigen (HLA)-A19, HLA-B22 and HLA-DR9 increased the susceptibility of JIA-associated uveitis whereas HLA-DR1 was protective for uveitis development [8]. Other HLA alleles have also been reported to be associated with JIA-associated uveitis such as HLA-DRw5, HLA-DRB1*1104, HLA-DRB1*1301 [8]–[10]. It is suggested that the genetic factors play important role in pediatric uveitis and investigating proper genetic factors is imperative for pediatric uveitis.

MicroRNAs (miRNAs) are endogenous ∼22 nt non-coding RNA molecules that function as negative regulators by targeting mRNAs for cleavage or translational repression, playing critical roles in diverse biologic processes, such as infection, immune response, inflammation and tumorigenesis [11], [12]. The microRNA-146a (miR-146a) was reported as a negative regulator of innate immunity in systemic lupus erythematosus (SLE) patients and a key negative regulator of inflammation [13], [14]. It was also indicated to be a vital regulator during viral infection [15], [16].

The Ets1 transcription factor is a member of the Ets gene family and is highly conserved throughout evolution. It is known to regulate a number of important biological processes in normal cells and in tumors and associated with regulation of immune cell function and with an aggressive behavior in tumors [19], [20]. Moreover, Ets1 can bind to the miR-146a promoter region and markedly affects miR-146a promoter activity in vitro. Knockdown of Ets-1 impaired the induction of miR-146a, whereas overexpression of Ets-1 enhanced the induction of miR-146a [21].

Single nucleotide polymorphisms (SNPs), located either in the pre-miRNAs or within miRNA-binding sites, have been shown to affect miRNA target expression, thereby possibly contributing to disease susceptibility [17], [18]. Luo et al. identified 12 variants of miR-146a, 9 of which were already reported in the dbSNP database Build 130 [21]. Three variants of these 9 had a minor allele frequency (MAF)>1% (rs2910164, rs57095329, and rs6864584). A recent study showed that SNPs rs1128334 and rs10893872 located in the 3′UTR of Ets-1 were on putative miRNA binding sites and were both associated with SLE in Asian populations [22].

Considering the role of miR-146a in the development of autoimmune diseases and the role of Ets-1 as a regulator of miR-146a expression, we investigated the association of miR-146a/rs2910164, miR-146a/rs57095329, miR-146a/rs6864584, ets-1/rs1128334 and ets-1/rs10893872 with pediatric uveitis in a Han Chinese population. We showed a significant association between both miR-146a and Ets-1 gene polymorphisms and pediatric uveitis in our study population.

## Materials and Methods

### Study population

The study group comprised 520 consecutive Han Chinese pediatric uveitis patients and 1204 unselected, consecutive, ethnically and geographically matched normal controls. The blood samples of patients and controls were obtained from the Uveitis Study Center of the Sun Yat-sen University (Guangzhou, P.R. China) and the First Affiliated Hospital of Chongqing Medical University (Chongqing, P.R. China). Pediatric uveitis was defined as uveitis first occurring in a child under 16 years old. Children with Behcet's disease, Vogt-Koyanagi-Harada syndrome or with definite infectious uveitis were excluded. JIA was defined as arthritis of unknown etiology presenting in children younger than 16 years old and persisting for at least 6 weeks according to the criteria of International League of Associations for Rheumatology [23]. The study was approved by the Local Ethics Research Committee of the First Affiliated Hospital of Chongqing Medical University. The written informed consent from the guardians on the behalf of minor participants and adult subjects involved in the study were obtained. We adhered to the tenets of the Declaration of Helsinki during all procedures of this study.

### Clinical and laboratory observations

Pediatric uveitis patients underwent a uveitis screening protocol encompassing ANA (n = 34; positive rate was 17.8%), RF (n = 32; positive rate was 15.5%), and FFA (n = 192; microvascular leakage rate detected by FFA was found in 62.1% of tested patients). An ANA titer above 1∶100 and an RF level above 20.0 IU/ml were considered positive. FFA was considered a positive result when dye leakage from a retinal vessel was observed. All these tests were performed in the First Affiliated Hospital of Chongqing Medical University (Chongqing, P.R. China).

### DNA extraction and genotyping

Genomic DNA of patients and healthy controls was extracted by the QIAamp DNA Blood Mini Kit (Qiagen, Hilden, Germany). Amplification of the target DNA sequence was analyzed by polymerase chain reaction (PCR) and the primers used herein are shown in Table 1. Each PCR reaction was carried out in a 10 µl reaction volume containing 5 µl Premix Taq (Ex Taq Version; TaKaRa Biotechnology Co. Ltd., Dalian, China), 20 pmol primers, 0.2 µg genomic DNA and proper sterile purified water. Genotyping of the five tested SNPs was carried out by PCR-restriction fragment length polymorphism (RFLP) analysis. PCR products were digested with 2 U of restriction enzymes which are listed in Table 1. The digestion products were separated on 4% agarose gels and stained with GoldView (SBS Genetech Beijing, China). Moreover, 10% of the samples were randomly selected to confirm the PCR-RFLP results using direct sequencing (Invitrogen Biotechnology Co., Guangzhou, China).

**Table 1:** Primers and restriction enzymes used in RFLP analysis.

SNP	Primers	Tm (°C)	Restriction enzymes
rs2910164	5′-ATGGGTTGTGTCAGTGTCAGACAT-3′	58	HSP92II
	5′-TGCCTTCTGTCTCCAGTCTTCCAA-3′		
rs17057381	5′-GTGCTCAGTTACTGTCCATGCACTT-3′	60	AflII
	5′-CAGGCATAGAGGAAGGGGAGATTA-3′		
rs57095329	5′-GGGGCTGCGGAGAGTACCG-3′	60	MspI
	5′-GGACCCTCTTGCAGCACGTGTC-3′		
rs6864584	5′-CGATAAAGCTCTCGGGATTTC-3′	56	ApaI
	5′-TCTTATTTGCTGGGGTAGAGGA-3′		
rs1128334	5′-TATTGTGTTTGACTATTTTCCAACAT-3′	55	HSP92II
	5′-ACTTACATCGCTACATCTCT-3′		
rs10893872	5′-ATCCCAGACCAAACCCAGTA-3′	60	TSP509I
	5′-TGGGCAGTAACAGGCTCTTT-3′		

RFLP, restriction fragment length polymorphism; SNP, single nucleotide polymorphism.

### RNA extraction and real-time RCR

Peripheral blood mononuclear cells (PBMCs) were isolated from venous blood of healthy controls by Ficoll-Hypaque density-gradient centrifugation. Total RNA was extracted from PBMCs using TRIzol (Invitrogen, San Diego, California, USA), and then it was reverse transcribed by transcriptase kit (Applied Biosystems, ABI, Foster City, California, USA). Real-time PCR was performed to detect the quantity of Ets-1 mRNA using the Applied Biosystems 7500 Real-Time PCR System (Applied Biosystems, Foster City, CA) with the following primers Ets-1F: 5′-GCAGCCAGTCATCTTTCAACAGCC-3′; Ets-1R: 5′-TCAGCACGGTCCCGCACATA-3′. The β-actin (β-actinF: 5′- GGATGCAGAAGGAGATCACTG-3′ β-actinR: 5′-CGATCCACACGGAGTACTTG-3′) and GAPDH (F: 5′-GGGTGTGAACCATGAGAAGT-3′; R: 5′-GGCATGGACTGTGGTCATGA-3′) were chosen as dual internal reference genes to normalize the expression of Ets-1. Relative expression levels were calculated using the 2^−ΔΔCt^ method.

### Statistical analysis

A Hardy–Weinberg equilibrium (HWE) calculation was performed to ensure a balance in genotype and allele distribution between patients and normal controls. HWE was tested in the subjects using the χ^2^ test. Genotype and allele frequencies were compared between patients and controls by χ^2^ test using SPSS version 17.0 (SPSS, Inc., Chicago, IL). The P values were corrected (Pc) with the Bonferroni correction by multiplying with the number of analyses performed. The odds ratio (OR) was used to determine whether a particular SNP is a risk factor for pediatric uveitis patients, and to compare the magnitude of various SNPs for pediatric uveitis patients (OR = 1: Exposure does not affect odds of pediatric uveitis; OR>1: Exposure associated with higher odds of pediatric uveitis; OR<1: Exposure associated with lower odds of pediatric uveitis) [24]. The independent-samples T test was used to compare Ets-1 expression among three genotype groups. P values <0.05 were considered to be statistically significant.

## Results

### Clinical features of pediatric uveitis patients and normal controls

Detailed clinical findings of the enrolled pediatric uveitis patients and normal controls were presented in Table 2. Age and gender distribution in pediatric uveitis patients and normal controls were also described in Table 2. Only category of JIA-associated uveitis (n = 70) can be confirmed in our study. Other cases cannot be confirmed in which category of pediatric uveitis.

**Table 2:** Age, gender and clinical features distribution in pediatric uveitis patients and controls.

Clinical features	%	
Pediatric uveitis patients	n = 520	
Age	9.6±3.6	
Male	242	46.5
Female	278	53.5
Uveitis	520	100
Pediatric uveitis with JIA	70	13.5
FFA(+)	192(309 tested)	62.1
ANA(+)	34(191 tested)	17.8
RF(+)	32(206 tested)	15.5
Controls	n = 1204	
Mean age	34.8±11.4	
Male	545	45.3
Female	659	54.7

JIA, juvenile idiopathic arthritis; FFA, fundus fluorescein angiography; ANA, anti-nuclear antigen; RF, rheumatoid factor.

### Genotype and allele frequencies of SNPs in patients and controls

A total of 5 SNPs of miR-146a and Ets-1 were successfully genotyped in 520 pediatric uveitis patients and 1204 normal controls. The distribution of genotype frequencies of 5 SNPs were accorded with HWE in the controls. Altogether we found two SNPs (rs2910164 and rs10893872) that were associated with pediatric uveitis (Table 3). The frequencies of the rs2910164 CC genotype and C allele in pediatric uveitis patients were significantly lower than that in normal controls (P = 8.93×10^−5^, P_c_ = 0.001, OR = 0.646; P = 5.50×10^−7^, P_c_ = 2.75×10^−6^, OR = 0.686, respectively). While the frequencies of the rs2910164 GG genotype and G allele were significantly higher in pediatric uveitis patients compared to normal controls (P = 2.07×10^−5^, P_c_ = 3.11×10^−4^, OR = 1.773; P = 5.50×10^−7^, P_c_ = 2.75×10^−6^, OR = 1.457, respectively). In SNP rs10893872, the frequencies of the CC genotype and C allele were significantly increased (P = 2.59×10^−5^, P_c_ = 3.89×10^−4^, OR = 1.582; P = 0.002, P_c_ = 0.01, OR = 1.259) in patients compared to controls while the CT genotype and T allele were significantly decreased in patients compared to controls (P = 2.63×10^−4^, P_c_ = 0.004, OR = 0.680; P = 0.002, P_c_ = 0.01, OR = 0.794). There was no statistically significant difference concerning the genotype and allele of the other three SNPs between pediatric uveitis patients and controls (Table 3).

**Table 3:** Frequencies of genotypes and alleles of microRNA (miR)-146a and Ets-1 polymorphisms in pediatric uveitis patients and controls.

SNP	Genotyp Allele	Pediatric uveitis (n = 520)	Controls (n = 1204)	χ2	p value	pc value	OR (95% CI)
rs2910164	GG	113(0.217)	163(0.135)	18.127	2.07×10^−5^	3.11×10^−4^	1.773 (1.359–2.313)
	GC	248(0.477)	553(0.459)	0.453	0.501	NS	1.073(0.873–1.319)
	CC	159(0.306)	488(0.405)	15.350	8.93×10^−5^	0.001	0.646(0.519–0.805)
	G	474(0.456)	879(0.365)	25.080	5.50×10^−7^	2.75×10^−6^	1.457(1.257–1.688)
	C	566(0.544)	1529(0.635)	25.080	5.50×10^−7^	2.75×10^−6^	0.686(0.592–0.796)
rs57095329	AA	317(0.609)	801(0.665)	4.937	0.026	NS	0.786(0.635–0.972)
	AG	184(0.354)	372(0.309)	3.347	0.067	NS	1.225(0.985–1.522)
	GG	19(0.037)	31(0.026)	1.502	0.220	NS	1.435(0.803–2.564)
	A	818(0.787)	1974(0.820)	5.206	0.023	NS	0.810(0.676–0.971)
	G	222(0.213)	434(0.180)	5.206	0.023	NS	1.234 (1.030–1.479)
rs6864584	CC	2(0.004)	5(0.004)	0.008	0.927	NS	0.926(0.179–4.788)
	CT	39(0.075)	112(0.093)	1.476	0.224	NS	0.791(0.541–1.156)
	TT	479(0.921)	1087(0.903)	1.466	0.226	NS	1.257(0.867–1.823)
	C	43 (0.041)	122(0.051)	1.384	0.239	NS	0.808(0.566–1.153)
	T	997(0.959)	2286(0.949)	1.384	0.239	NS	1.237(0.867–1.766)
rs1128334	AA	64(0.123)	145(0.120)	0.024	0.877	NS	1.025(0.749–1.403)
	AG	253(0.487)	544(0.452)	1.760	0.185	NS	1.150(0.936–1.413)
	GG	203(0.390)	515(0.428)	2.085	0.149	NS	0.857(0.694–1.057)
	A	381(0.366)	834(0.346)	1.273	0.259	NS	1.091(0.938–1.270)
	G	659(0.634)	1574(0.654)	1.273	0.259	NS	0.916(0.788–1.066)
rs10893872	CC	209(0.402)	359(0.298)	17.695	2.59×10^−5^	3.89×10^−4^	1.582(1.277–1.960)
	CT	218(0.419)	620(0.515)	13.320	2.63×10^−4^	0.004	0.680(0.552–0.837)
	TT	93(0.179)	225(0.187)	0.156	0.693	NS	0.948(0.726–1.238)
	C	636(0.612)	1338(0.556)	9.270	0.002	0.01	1.259(1.085–1.460)
	T	404(0.388)	1070(0.444)	9.270	0.002	0.01	0.794(0.685–0.921)

pc, Bonferroni corrected p value; OR, odds ratio; NS, not significant; SNP, single nucleotide polymorphism.

We subsequently studied the associations of the 5 SNPs with clinical and laboratory observations of pediatric uveitis including FFA, RF and ANA. An association was found between rs2910164 and patients with microvascular leakage as detected by FFA (FFA^+^). The GG genotype and G allele frequencies of rs2910164 were significantly increased in FFA^+^ patients compared to controls (P = 6.84×10^−4^, P_c_ = 0.01, OR = 1.899; P = 9.41×10^−4^, P_c_ = 0.005, OR = 1.441) while the C allele frequency was significantly decreased (P = 9.41×10^−4^, P_c_ = 0.005, OR = 0.694) (Table 4). No statistically significant association was found between the other four SNPs and FFA^+^ patients. Moreover, there was no statistically significant association between the five SNPs and patient groups that were subdivided according to their RF or ANA status or whether they had JIA or not (data not shown).

**Table 4:** Genotype and allele frequency analysis of rs2910164 polymorphism in FFA positive pediatric uveitis patients.

SNP	Genotyp Allele	FFA^+^ pediatric uveitis (n = 192)	Controls (n = 1204)	χ2	p value	pc value	OR (95% CI)
rs2910164	GG	44(0.229)	163(0.135)	11.533	6.84×10^−4^	0.01	1.899(1.305–2.762)
	GC	86(0.448)	553(0.459)	0.086	0.769	NS	0.955(0.703–1.297)
	CC	62(0.323)	488(0.405)	4.709	0.030	NS	0.700(0.506–0.967)
	G	174(0.453)	879(0.365)	10.941	9.41×10^−4^	0.005	1.441(1.160–1.791)
	C	210(0.547)	1529(0.635)	10.941	9.41×10^−4^	0.005	0.694(0.558–0.862)

pc, Bonferroni corrected p value; OR, odds ratio; NS, not significant; SNP, single nucleotide polymorphism.

### The influence of rs10893872 on Ets-1 expression

**Figure 1:**
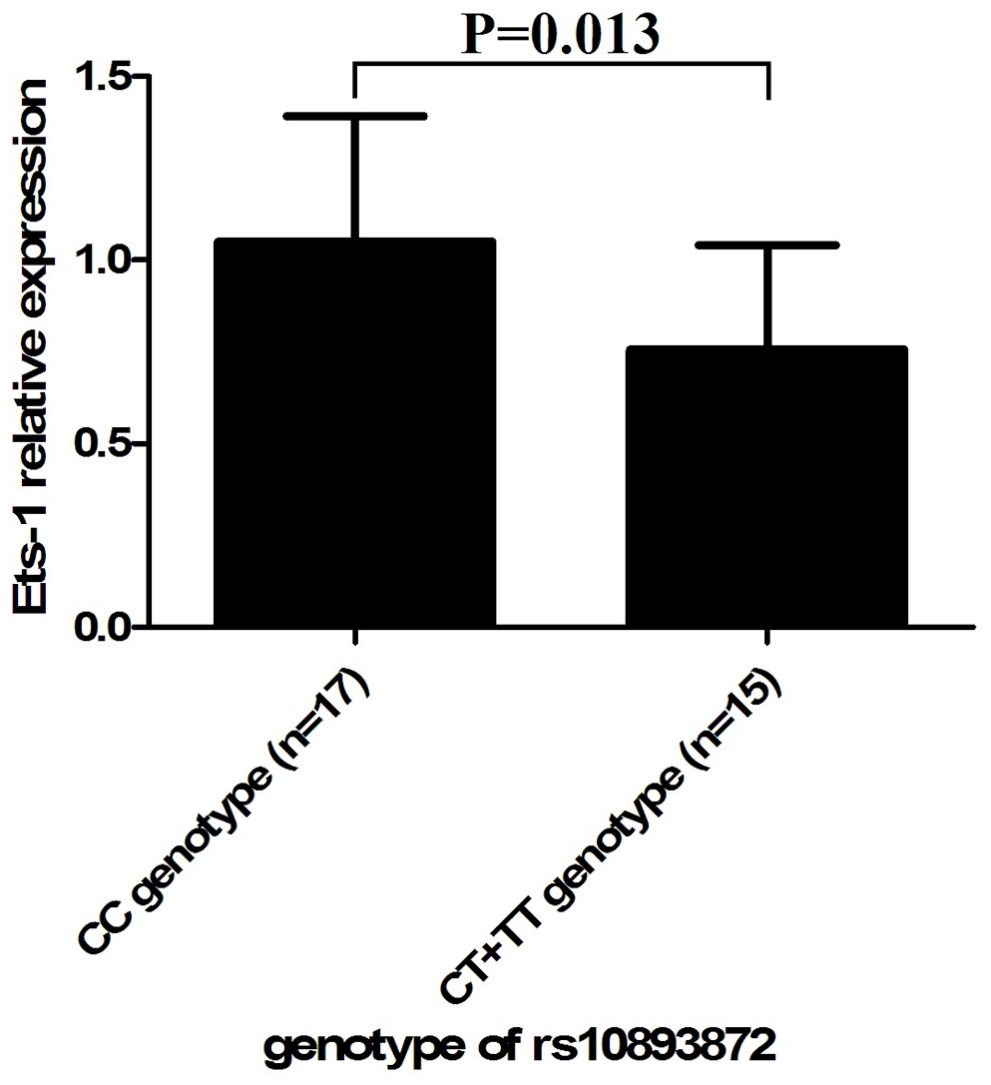
The relative expression of Ets-1. Ets-1 expression with three different genotypes of single nucleotide polymorphism (SNP) rs10893872 in peripheral blood mononuclear cells obtained from genotyped healthy controls (PBMCs). Real-time PCR analysis of Ets-1 expression in PBMCs derived from healthy individuals of SNP rs10893872 CC, CT and TT genotypes. The y axis represents relative Ets-1 expression level by real-time PCR of each genotype of SNP rs10893872. Data are shown as mean±SD.

Because a significant association was found between SNP rs10893872 and pediatric uveitis patients, we further tested the expression of Ets-1 in PBMCs obtained from 32 healthy individuals with a known genotype for this SNP. The mean expression of Ets-1 in CC carriers was 1.39-fold higher than that in CT and TT carriers (P = 0.013) (Figure 1).

## Discussion

In the present study, we investigated the association of five SNPs of miR-146a and the transcription factor Ets-1 with pediatric uveitis patients in a Chinese Han population. To the best of our knowledge, this is the first study to investigate the possible associations of miR-146a and Ets-1 polymorphisms with pediatric uveitis. It was found that rs2910164 of miR-146a and rs10893872 of Ets-1 were both associated with pediatric uveitis. The expression of Ets-1 in individuals carrying the rs10893872 CC genotype was increased compared to the CT and TT genotype, suggesting a functional explanation for the observed association.

The frequencies of the rs2910164 CC genotype and C allele were significantly decreased in patients as compared to normal controls while the GG genotype and G allele were significantly increased. It was suggested that miR-146a might be a susceptibility factor for pediatric uveitis. Recently, we studied the miR-146a rs2910164 polymorphisms in acute anterior uveitis patients with ankylosing spondylitis and found a similar association as shown in the present work for pediatric uveitis patients [25]. Several similar results were also reported in ocular Behçet's disease [26], asthma [27] and various types of carcinoma [28]–[30]. Nevertheless, three SNPs of miR-146a/rs2910164, ets-1/rs1128334, and ets-1/rs10893872 were genotyped in Han Chinese patients with Fuchs uveitis syndrome (FUS), and no significant difference could be found between patients and the normal controls [31]. This is different with results found herein. Moreover, afunctional analysis performed in our previous study showed that carriers of the protective CC genotype showed a decreased expression of miR-146a and certain proinflammatory cytokines such as IL-17, TNF-α and IL-1β [26]. These results suggest that the rs2910164 CC genotype and C allele and GG genotype and G allele are common predisposing factors for pediatric uveitis despite the fact that the pathogenic mechanisms underlying cancer and pediatric uveitis are totally different.

We also identified an association between SNP rs10893872 of Ets-1 and pediatric uveitis in the present study. The CC genotype and C allele frequencies of this SNP were significantly increased in patients compared to controls while the frequencies of the CT genotype and T allele were significantly decreased, indicating that the CC genotype and C allele were predisposing factors for pediatric uveitis. Based on this association, we further designed a study to investigate whether this SNP (rs10893872) affected the expression of Ets-1. Our study demonstrated that the Ets-1 expression was significantly upregulated in healthy individuals carrying the CC genotype of rs10893872. We performed this assay in healthy individuals since immunosuppressive treatment in the patient group could possibly alter gene expression. SNP rs10893872 of Ets-1 was found to be highly associated with SLE [22], [32]. Differently, there was no significant association of SNP rs10893872 of Ets-1 found in ocular Behçet's disease, Vogt–Koyanagi–Harada syndrome, and Fuchs uveitis syndrome in Chinese Han patients with previous works [26], [31].

The transcription factor Ets-1, originally discovered as an oncogene (v-ets) within the genome of the avian leukemia virus [33], shows a dual nature in autoimmune diseases [34]. It was reported to be overexpressed in rheumatoid arthritis (RA) synovial membrane and to be involved in the destructive pathway of RA [35]. Nevertheless, the expression of Ets-1 in SLE was lower in PBMCs as compared with that of healthy subjects [36]. Ets-1 also plays important roles in regulating the differentiation of T helper cell subsets, cytotoxic T cells, B cells and other cell types, and controlling the expression of cytokine and chemokine genes in a wide variety of different cellular contexts [37]. The regulatory T cell (Treg) lineage is a key player maintaining peripheral self-tolerance and modulating almost any type of immune responses. Julia K. Polansky *et. al.* have proposed that Ets-1 binds to the CpG-rich Treg specific demethylated region only in its demethylated state, thereby restricting stable Foxp3 expression to the Treg lineage [38]. Concerning the important roles of Ets-1, the increased frequency of the rs10893872 CC genotype in patients and the increased expression of Ets-1 in CC genotype carriers suggest that Ets-1 is a predisposing factor in pediatric uveitis.

It is worthwhile to mention that there are several limitations in the present study. The sample of patients in our study is relatively small and only Han Chinese cohorts are included. Therefore, the results observed in this study need to be confirmed using a large sample size and should include other ethnic populations. We have not investigated whether the SNP rs10893872 influencing Ets-1 expression can also affect the production of cytokines importantly involved in pediatric uveitis development. Further studies are needed to clarify this issue.

In conclusion, our study has identified the associations of rs2910164 (miR-146a) and rs10893872 (Ets-1) with pediatric uveitis. Furthermore, our study has suggested that SNP rs10893872 may affect the genetic predisposition to this disease possibly through modulating the expression of Ets-1.

## References

[1] Babu K, Mahendradas P (2013) Medical Management of Uveitis–Current Trends. Indian journal of ophthalmology 61: 277. 23803479 10.4103/0301-4738.114099 PMC3744780

[2] Clarke LA, Guex-Crosier Y, Hofer M (2013) Epidemiology of uveitis in children over a 10-year period. Clin Exp Rheumatol 31: 633–637. 23432940

[3] Lerman MA, Burnham JM, Chang PY, Daniel E, Foster CS, et al (2013) Response of pediatric uveitis to tumor necrosis factor-alpha inhibitors. J Rheumatol 40: 1394–1403. 23818712 10.3899/jrheum.121180 PMC3802519

[4] Cunningham J, Emmett T (2000) Uveitis in children. Ocular immunology and inflammation 8: 251–261. 11262655 10.1076/ocii.8.4.251.6459

[5] Päivönsalo-Hietanen T, Tuominen J, Matti Saari K (2000) Uveitis in children: Population-based study in Finland. Acta ophthalmologica Scandinavica 78: 84–88. 10726797 10.1034/j.1600-0420.2000.078001084.x

[6] Smith JA, Mackensen F, Sen H, Leigh JF, Watkins AS, et al (2009) Epidemiology and course of disease in childhood uveitis. Ophthalmology 116: 1544–e1541, 1544-1551, e1541. 19651312 10.1016/j.ophtha.2009.05.002 PMC2937251

[7] Tugal-Tutkun I (2011) Pediatric uveitis. Journal of Ophthalmic & Vision Research 6: 259. 22454749 PMC3306124

[8] Zulian F, Martini G, Falcini F, Gerloni V, Zannin ME, et al (2002) Early predictors of severe course of uveitis in oligoarticular juvenile idiopathic arthritis. The Journal of rheumatology 29: 2446–2453. 12415607

[9] Melin-Aldana H, Giannini EH, Taylor J, Lovell DJ, Levinson JE, et al (1992) Human leukocyte antigen-DRB1^*^ 1104 in the chronic iridocyclitis of pauciarticular juvenile rheumatoid arthritis. The Journal of pediatrics 121: 56–60. 1625093 10.1016/s0022-3476(05)82541-7

[10] Zeggini E, Packham J, Donn R, Wordsworth P, Hall A, et al (2006) Association of HLA-DRB1* 13 with susceptibility to uveitis in juvenile idiopathic arthritis in two independent data sets. Rheumatology 45: 972–974. 16495319 10.1093/rheumatology/kel049

[11] Bartel DP (2004) MicroRNAs: genomics, biogenesis, mechanism, and function. Cell 116: 281–297. 14744438 10.1016/s0092-8674(04)00045-5

[12] Bushati N, Cohen SM (2007) microRNA functions. Annu Rev Cell Dev Biol 23: 175–205. 17506695 10.1146/annurev.cellbio.23.090506.123406

[13] Boldin MP, Taganov KD, Rao DS, Yang L, Zhao JL, et al (2011) miR-146a is a significant brake on autoimmunity, myeloproliferation, and cancer in mice. The Journal of experimental medicine 208: 1189–1201. 21555486 10.1084/jem.20101823 PMC3173243

[14] Tang Y, Luo X, Cui H, Ni X, Yuan M, et al (2009) MicroRNA-146a contributes to abnormal activation of the type I interferon pathway in human lupus by targeting the key signaling proteins. Arthritis & Rheumatism 60: 1065–1075. 19333922 10.1002/art.24436

[15] Hou J, Wang P, Lin L, Liu X, Ma F, et al (2009) MicroRNA-146a feedback inhibits RIG-I-dependent Type I IFN production in macrophages by targeting TRAF6, IRAK1, and IRAK2. The Journal of Immunology 183: 2150–2158. 19596990 10.4049/jimmunol.0900707

[16] Cameron JE, Yin Q, Fewell C, Lacey M, McBride J, et al (2008) Epstein-Barr virus latent membrane protein 1 induces cellular MicroRNA miR-146a, a modulator of lymphocyte signaling pathways. Journal of virology 82: 1946–1958. 18057241 10.1128/JVI.02136-07 PMC2258704

[17] Clop A, Marcq F, Takeda H, Pirottin D, Tordoir X, et al (2006) A mutation creating a potential illegitimate microRNA target site in the myostatin gene affects muscularity in sheep. Nature genetics 38: 813–818. 16751773 10.1038/ng1810

[18] Yu Z, Li Z, Jolicoeur N, Zhang L, Fortin Y, et al (2007) Aberrant allele frequencies of the SNPs located in microRNA target sites are potentially associated with human cancers. Nucleic acids research 35: 4535–4541. 17584784 10.1093/nar/gkm480 PMC1935019

[19] Dittmer J (2003) The biology of the Ets1 proto-oncogene. Mol Cancer 2: 29. 12971829 10.1186/1476-4598-2-29 PMC194255

[20] Garrett-Sinha LA (2013) Review of Ets1 structure, function, and roles in immunity. Cell Mol Life Sci 70: 3375–3390. 23288305 10.1007/s00018-012-1243-7 PMC3636162

[21] Luo X, Yang W, Ye D-Q, Cui H, Zhang Y, et al (2011) A functional variant in microRNA-146a promoter modulates its expression and confers disease risk for systemic lupus erythematosus. PLoS Genetics 7: e1002128. 21738483 10.1371/journal.pgen.1002128 PMC3128113

[22] Yang W, Shen N, Ye D-Q, Liu Q, Zhang Y, et al (2010) Genome-wide association study in Asian populations identifies variants in ETS1 and WDFY4 associated with systemic lupus erythematosus. PLoS Genetics 6: e1000841. 20169177 10.1371/journal.pgen.1000841 PMC2820522

[23] Petty RE, Southwood TR, Manners P, Baum J, Glass DN, et al (2004) International League of Associations for Rheumatology classification of juvenile idiopathic arthritis: second revision, Edmonton, 2001. The Journal of rheumatology 31: 390. 14760812

[24] Szumilas M (2010) Explaining odds ratios. Journal of the Canadian Academy of Child and Adolescent Psychiatry 19: 227. 20842279 PMC2938757

[25] Qi J, Hou S, Zhang Q, Liao D, Wei L, et al (2013) A functional variant of pre-miRNA-196a2 confers risk for Behcet's disease but not for Vogt–Koyanagi–Harada syndrome or AAU in ankylosing spondylitis. Human genetics 1–10. 23928854 10.1007/s00439-013-1346-8

[26] Zhou Q, Hou S, Liang L, Li X, Tan X, et al (2012) MicroRNA-146a and Ets-1 gene polymorphisms in ocular Behçet's disease and Vogt–Koyanagi–Harada syndrome. Annals of the rheumatic diseases 10.1136/annrheumdis-2012-201627 23268366

[27] Jiménez-Morales S, Gamboa-Becerra R, Baca V, Río-Navarro D, López-Ley D, et al (2012) MiR-146a polymorphism is associated with asthma but not with systemic lupus erythematosus and juvenile rheumatoid arthritis in Mexican patients. Tissue Antigens 9999. 10.1111/j.1399-0039.2012.01929.x 22823586

[28] Guo H, Wang K, Xiong G, Hu H, Wang D, et al (2010) A functional varient in microRNA-146a is associated with risk of esophageal squamous cell carcinoma in Chinese Han. Familial cancer 9: 599–603. 20680470 10.1007/s10689-010-9370-5

[29] Jazdzewski K, Murray EL, Franssila K, Jarzab B, Schoenberg DR, et al (2008) Common SNP in pre-miR-146a decreases mature miR expression and predisposes to papillary thyroid carcinoma. Proceedings of the National Academy of Sciences 105: 7269–7274. 10.1073/pnas.0802682105 PMC2438239 18474871

[30] Zhou F, Zhu H, Luo D, Wang M, Dong X, et al (2012) A Functional Polymorphism in Pre-miR-146a Is Associated with Susceptibility to Gastric Cancer in a Chinese Population. DNA and Cell Biology 31: 1290–1295. 22455393 10.1089/dna.2011.1596

[31] Zhou Q, Kijlstra A, Hou S, Yu H, Zhang X, et al (2012) Lack of association of miR-146a and Ets-1 gene polymorphisms with Fuchs uveitis syndrome in Chinese Han patients. Mol Vis 18: 426–430. 22355253 PMC3283209

[32] Zhang J, Zhang Y, Zhang L, Yang J, Ying D, et al (2013) Epistatic Interaction between Genetic Variants in Susceptibility Gene ETS1 Correlates with IL-17 Levels in SLE Patients. Annals of human genetics 10.1111/ahg.12018 23614478

[33] Nunn MF, Seeburg PH, Moscovici C, Duesberg PH (1983) Tripartite structure of the avian erythroblastosis virus E26 transforming gene. 10.1038/306391a0 6316155

[34] Leng R-X, Pan H-F, Chen G-M, Feng C-C, Fan Y-G, et al (2011) The dual nature of Ets-1: focus to the pathogenesis of systemic lupus erythematosus. Autoimmunity reviews 10: 439–443. 21296190 10.1016/j.autrev.2011.01.007

[35] Redlich K, Kiener HP, Schett G, Tohidast-Akrad M, Selzer E, et al (2001) Overexpression of transcription factor Ets-1 in rheumatoid arthritis synovial membrane: Regulation of expression and activation by interleukin-1 and tumor necrosis factor α. Arthritis & Rheumatism 44: 266–274. 11229456 10.1002/1529-0131(200102)44:2<266::AID-ANR43>3.0.CO;2-G

[36] LI Y, SUN L-D, LU W-S, HU W-L, GAO J-P, et al (2010) Expression analysis of ETS1 gene in peripheral blood mononuclear cells with systemic lupus erythematosus by real-time reverse transcription PCR. Chinese medical journal 123: 2287–2288. 20819682

[37] Russell L, Garrett-Sinha LA (2010) Transcription factor Ets-1 in cytokine and chemokine gene regulation. Cytokine 51: 217–226. 20378371 10.1016/j.cyto.2010.03.006

[38] Polansky JK, Schreiber L, Thelemann C, Ludwig L, Krüger M, et al (2010) Methylation matters: binding of Ets-1 to the demethylated Foxp3 gene contributes to the stabilization of Foxp3 expression in regulatory T cells. Journal of molecular medicine 88: 1029–1040. 20574810 10.1007/s00109-010-0642-1 PMC2943068

